# Keep It Simple: Using README Files to Advance Standardization in Chronobiology

**DOI:** 10.3390/clockssleep5030033

**Published:** 2023-08-30

**Authors:** Tomasz Zieliński, James J. L. Hodge, Andrew J. Millar

**Affiliations:** 1 Centre for Engineering Biology, School of Biological Sciences, University of Edinburgh, Edinburgh EH9 3JD, UK; andrew.millar@ed.ac.uk; 2 School of Physiology, Pharmacology and Neuroscience, University of Bristol, Bristol BS8 1TD, UK; james.hodge@bristol.ac.uk

**Keywords:** FAIR, circadian data, chronobiology, standardization, metadata

## Abstract

Standardization plays a crucial role in ensuring the reliability, reproducibility, and interoperability of research data in the biomedical sciences. Metadata standards are one foundation for the FAIR (Findable, Accessible, Interoperable, and Reusable) principles of data management. They facilitate data discovery, understanding, and reuse. However, the adoption of metadata standards in biological research lags in practice. Barriers such as complexity, lack of incentives, technical challenges, resource constraints, and resistance to change hinder widespread adoption. In the field of chronobiology, standardization is essential but faces particular challenges due to the longitudinal nature of experimental data, diverse model organisms, and varied measurement techniques. To address these challenges, we propose an approach that emphasizes simplicity and practicality: the development of README templates tailored for particular data types and species. Through this opinion article, our intention is to initiate a dialogue and commence a community-driven standardization process by engaging potential contributors and collaborators.

In this opinion article, we aim to present a straightforward approach to metadata standardization within the field of chronobiology, centred around the usage of text templates for data description. Our goal is to initiate a discourse and instigate a community-driven standardization process by engaging contributors and collaborators. 

Metadata standards serve as a crucial foundation for the FAIR (Findable, Accessible, Interoperable, and Reusable) principles [1], playing a vital role in enabling efficient data management and utilization. Comprehensive data descriptions, also known as metadata, supported by metadata standards facilitate understanding of the data’s content and context, enabling efficient discovery and reuse [2,3]. The achievement of interoperability, a key aspect of FAIR, relies on the use of common vocabularies, ontologies, and formats specified using metadata standards. These standardized structures allow for seamless integration of data from diverse sources, promoting cross-domain collaborations and knowledge integration [4]. Moreover, the machine-readability of formalized metadata enables automated processing and integration, further streamlining data analysis and interpretation.

In the field of biomedical sciences, standardization plays a crucial role in ensuring the reliability, reproducibility, and interoperability of research data [5,6]. Establishing guidelines and protocols for data collection and descriptions significantly enhances the quality of scientific investigations. By adhering to standardized metadata practices, researchers can gather consistent and systematic information across studies, minimizing biases and errors. Such standardization also enables reliable comparisons, facilitates meta-analyses, and allows for data pooling from multiple sources, ultimately leading to more generalizable findings and potential discoveries [7,8]. Embracing common metadata standards enables researchers to provide detailed information about their data, which is crucial for interpretation and contextualization.

While various standards and guidelines have been developed within biological communities to address data standardization [9], adoption rates vary across different fields and research communities [10,11]. The Minimum Information about a Biological or Biomedical Investigation (MIBBI) umbrella [12], for example, collects valuable guidelines including Minimum Information About a Microarray Experiment (MIAME) [13], Minimum Information about any (X) Sequence (MIxS) [14], Minimum Information About a Proteomics Experiment (MIAPE) [15], Minimum Information About a Simulation Experiment (MIASE) [16], and Minimum Information for Publication of Quantitative Real-Time PCR Experiments (MIQE) [17], among others. These guidelines are often developed with extensive input from their respective communities of practice. Despite their ready availability, practical adoption of these standards often lags behind. Many MIBBI guidelines are currently only available in their original publications, with documentation, templates, and example files no longer accessible online, when we searched in 2023. This discrepancy highlights the disconnection between standardization efforts and their practical adoption within the research community.

Several barriers hinder the widespread adoption of standards [18]:

- Extra effort: Implementing metadata standards and guidelines may be perceived as complex and time-consuming. Software tools can help researchers prepare standards-compliant metadata, but writing and updating such tools is a further effort;
- Lack of incentives and recognition: if adherence to metadata standards is not incentivized or recognized by the scientific community, researchers may consider them additional burdensome requirements;
- Technical challenges: adopting metadata standards may require modifications to existing data management systems and infrastructures;
- Resource constraints: researchers may lack the necessary resources, including funding, technical expertise, and training, to effectively implement metadata standards, let alone the associated software tools and repositories;
- Resistance to change: researchers may be comfortable with their current data management practices and reluctant to adopt new standards.

Based on our experience, the best-adopted metadata standards tend to be supported by scientific publishers that mandate data deposition in repositories specifically designed to support these standards. The crucial elements are the enforcement by publishers and the software infrastructure that simplifies the metadata documentation and assures adherence to the standards [19]. However, the most widely adopted standards relate to molecular structure or cellular composition, rather than to a biological function such as rhythmicity.

In addition, we believe that the existing minimal information guidelines tend to prioritize technical aspects of measurement and experiment reproducibility, rather than emphasizing the reusability of generated output. For example, the MIQE guideline predominantly covers aspects like standard curves, thermocycling parameters, or buffers while providing limited information about the biological context, such as organism/sample characteristics, which are to be recorded in one field: “Definition of experimental and control groups”. Researchers interested in reusing expression-level data are often more concerned with these contextual details than the intricacies of the calibration curve.

In the field of chronobiology, the standardization of data collection and sharing holds immense potential for advancing the field [20,21]. The longitudinal nature of experimental data in chronobiology introduces specific challenges [22,23], but it also offers a strong incentive to re-use data that are laborious to acquire. While data collection processes are often automated, there is considerable variation in the methods employed by different research groups, involving diverse devices, protocols, and assessment tools for measuring circadian parameters. Additionally, the study of chronobiology encompasses diverse model organisms, each with specific characteristics and applicable experimental techniques. The studies share some defining issues, such as describing the internal, biological phase of measurement (or sufficient information to infer that phase) in addition to the wall-clock time when it was made.

The variety of analysis methods available in the field of chronobiology constitutes another compelling argument for data sharing. For instance, the assessment of periodicity in genome-wide data can be carried out using tools such as eJTK [24], BooteJTK [25], ARSER [26], RAIN [27], or a basic cosinor implementation [28], each presenting distinct strengths and limitations [29,30]. To ensure consistency, it is often essential to re-analyse raw data, especially when combining results from diverse studies.

Our experience in metadata acquisition for circadian data sharing is based on our BioDare2 repository [31,32]. BioDare2 serves as a repository for circadian and biological data, offering a platform for both data sharing and period analysis. In the initial version, BioDare, a detailed description of experimental factors relating to light and temperature regimes, growth conditions, sample type and genotype was required. This information was captured using forms generated from metadata schema definitions, with a controlled vocabulary of terms. The datasets were unambiguously described and highly re-usable. However, the substantial effort demanded from data creators to provide this description posed a significant hurdle and impeded the adoption of BioDare. Disturbingly, we observed multiple instances where users re-used descriptions from unrelated datasets to bypass the dataset annotation process, resulting in records with inaccurate descriptions.

The current version of BioDare2 is much less formal and requires less detail (comprising free-text descriptions, species, authorship, and technique). This has proven attractive to a substantial user base (exceeding 1000 users) and resulted in a vast collection of datasets (over 10,000). While this reduction in metadata has improved data accessibility, it has concurrently diminished reusability. In truth, a majority of the datasets lack sufficient metadata to be understood without the relevant publication. Nevertheless, resources like BioDare2 serve to foster public data sharing and facilitate data reanalysis.

To address these challenges and promote the adoption of metadata standards in the field of chronobiology, we propose the following approach:

1. Agile development: In order to ensure the relevance and applicability of our recommendations, we will adopt an agile development approach. This means that we will generate frequent and actionable recommendations that can be easily incorporated into existing workflows and software infrastructure. By adopting an iterative approach, we can avoid the issue of obsolescence and ensure that our guidelines remain up to date with evolving practices and technologies;
2. Enhanced metadata descriptions: While existing minimal information guidelines focus on technical aspects of data measurement and reproducibility, we believe it is essential to emphasize the reporting of biological and environmental contexts for datasets. To achieve this, we will develop guidelines and provide examples for reporting important experimental factors such as light and temperature entrainment or drug interventions during experiments. By capturing these contextual details, we aim to facilitate data reuse and enable comprehensive interpretations by researchers;
3. Utilization of README templates: To simplify the process of capturing metadata, we propose the use of simple README templates in a human-readable format, such as plain text. These templates will provide researchers with a clear structure for capturing the required metadata without requiring specialized technical knowledge or software tools. README templates can seamlessly integrate into existing data organization practices and repositories. This approach accommodates various needs and contextual information while promoting flexibility and ease of use. Additionally, README documents can be easily version-controlled, allowing for collaborative and iterative changes to the metadata. This adaptability ensures that the value of README files remains intact regardless of the target data repository, whether it is a generic, data-agnostic repository like Zenodo [33] and Figshare [34], or a domain-specific resource like BioDare2 [31];
4. Tailored templates: Instead of developing a single comprehensive template, we recognize the need to create multiple templates tailored to specific organisms and experimental techniques. This approach simplifies template usage and resolves issues related to different terminologies used for describing humans compared to model organism data. For instance, human data are typically grouped in cohorts and described with demographics, while data from model organisms are often recorded as biological replicates and described with genotypes. By tailoring the templates, we can provide researchers with focused guidance that is relevant to their specific experimental contexts;
5. Syntax for automatic parsing and validation: While simple README templates offer advantages, we acknowledge the importance of machine-readability and interoperability. To address this, we propose developing a syntax that enables at least automatic parsing and validation of the text documents. For example, we suggest using specific characters, such as #, to distinguish between keys and their values. By incorporating machine-readable syntax, we enhance the interoperability and compatibility of the metadata with data processing systems and repositories. This approach ensures compatibility with evolving guidelines and facilitates potential conversion to more formal formats (e.g., JSON) if necessary;
6. Collaboration with Metadata4Wearables: To align our efforts and ensure compatibility and complementarity, we plan to collaborate with the Metadata4Wearables [35] community. This community focuses on standardizing actigraphy and light exposure data using JSON schemas. By collaborating with Metadata4Wearables, we can leverage their expertise and complement our ongoing initiatives to create a cohesive approach to metadata standardization in chronobiology;
7. Dedicated GitHub repository: In order to disseminate our work and gather feedback from the scientific community, we have established a dedicated GitHub repository (https://github.com/circadianmentalhealth/circadian-data-standards) (accessed on 25 August 2023) [36]. We strongly encourage readers to contribute their thoughts, offer insights, and provide feedback on the proposed plan or draft templates using the issue tracking system within the repository. This collaborative approach ensures that the standards we develop reflect the needs and perspectives of the broader scientific community;
8. Future steps: Our future work involves listing circadian variables for routine use and recommending analysis methods for their estimation. Additionally, we will focus on improved interoperability by suggesting suitable ontologies and closed vocabularies for formal data descriptions.

We plan to use the Markdown compatible format for README templates. This format utilizes Markdown formatting characters to distinguish between keys, values, and comments, providing improved readability within data repositories that support Markdown, such as GitHub (see Figure 1). All Markdown headings regardless of their level are treated as keys, the subsequent text as their values, and comments that guide template completion are encoded as blockquotes. Researchers who are not familiar with Markdown can still treat the templates as simple text documents, ensuring accessibility for all users. Researchers can tailor the templates to their specific needs and contextual information by simply adding new headings. This adaptability allows for capturing a wide range of metadata elements without being constrained by a rigid structure. At the same time, metadata processors like repositories or aggregation pipelines, can easily extract the values of the supported (or required) keys while ignoring the remaining description elements.

We propose the development of a collection of templates specific to each organism and technique. However, the existence of multiple templates could potentially create challenges for both users and maintainers of the metadata templates. Researchers may feel overwhelmed and confused when faced with a large number of templates to choose from. To address this issue, we will implement a simple decision tree that guides users to the appropriate template based on their specific requirements. This decision tree could be easily implemented as a web form on this project’s GitHub pages, providing a user-friendly interface for template selection.

Another potential problem that arises from having multiple templates is the need to update them consistently when changes to nomenclature occur. For instance, if we decide to change the term “Authors” to “Contributors” in all templates, manually editing each text document could be a laborious task prone to errors. To overcome this challenge, we will encode specific aspects of the metadata, such as administrative metadata, actigraphy metadata, mice sample descriptions, etc., in separate files. Each template will then be created by assembling the relevant parts according to a predefined build recipe. This process can be easily achieved using makefiles or similar software build tools, ensuring consistent updates across all templates and reducing the risk of errors. Simultaneously, the adoption of a unified collection of building blocks allows for the mapping of customized templates into a master data model, capable of encompassing them all.

The availability of metadata templates could influence operational practices and the data-sharing culture within the domain of chronobiology, in the near term. By embracing README templates, researchers can be confident in capturing pertinent metadata in the correct format. Unlike online repository forms, README files can be completed during the course of experimental work rather than only at the point of dataset deposition. This approach might also reduce the duplication of effort, based on the likelihood that experimentalists are already recording identical information using different mediums, such as lab notebooks or embedded within data files. Creating metadata within the research workflow, at the time of data acquisition, improves the quality and accuracy of the accumulated metadata. The text templates are self-explanatory, necessitating no supplementary training. These description files can be prepared in any text editor and require no specialized software for handling. If a deeper integration into existing infrastructure is required, implementation is relatively straightforward, as explained in the use case of the public repositories below. All these aspects collectively mitigate three of the barriers to standards adoption: additional effort, technical challenges, and resource constraints.

While the templates themselves may not directly address the lack of incentives, they hold the potential to transform the sharing culture. Reviewers could insist on template completion as a requirement for publication, akin to the manner in which they mandate minimal information checklists for RT-PCR or MS Proteomics data.

Once approved and adopted by the community, the README templates should be easy to integrate into circadian and sleep repositories such as BioDare2 [31] or The National Sleep Research Resource [37]. To facilitate this integration, we plan to develop Java and Python libraries for parsing these README templates into key-value pairs, which can then be effortlessly indexed by the repositories. Moreover, online repositories can straightforwardly display the proposed Markdown files in a “dataset view”, thanks to several suitable libraries available for the task [38,39]. Drawing from our experience with BioDare/BioDare2, we are confident that incorporating metadata-rich README files into a repository is significantly simpler to implement than the development of web forms and widgets required to capture the same information. We also suspect that users are more inclined to add details to README files throughout their work compared to entering them into online repository web forms during submission.

The standardization of data collection, curation, and sharing best practices in circadian research is crucial for advancing our understanding of biological clocks between model organisms and translating the impact of this research to health outcomes. As demonstrated by expansive population-wide biobank endeavours like the UK Biobank [40], meticulously documented and consistently aggregated data encompassing diverse data types have yielded breakthrough discoveries and medical advancements. Undertaking similar research, but on data amalgamated from disparate projects, is the pinnacle aspiration of the FAIR initiative. The development of circadian community standards represents an important stride on this transformative journey, amplifying data interoperability, comparability, and reproducibility.

The proposed, simple approach to metadata standardization prioritizes the ease of use and overcomes barriers such as perceived added effort, technical challenges, and resource constraints. This approach has the potential to bring about a near-term change in the working and sharing culture in the field of chronobiology. The newly MRC-funded Circadian Mental Health Network [41] invites collaboration and contributions to both support and propel these initiatives forward. To facilitate engagement, a range of contact channels is accessible through the project’s GitHub repository [36].

## Figures and Tables

**Figure 1:**
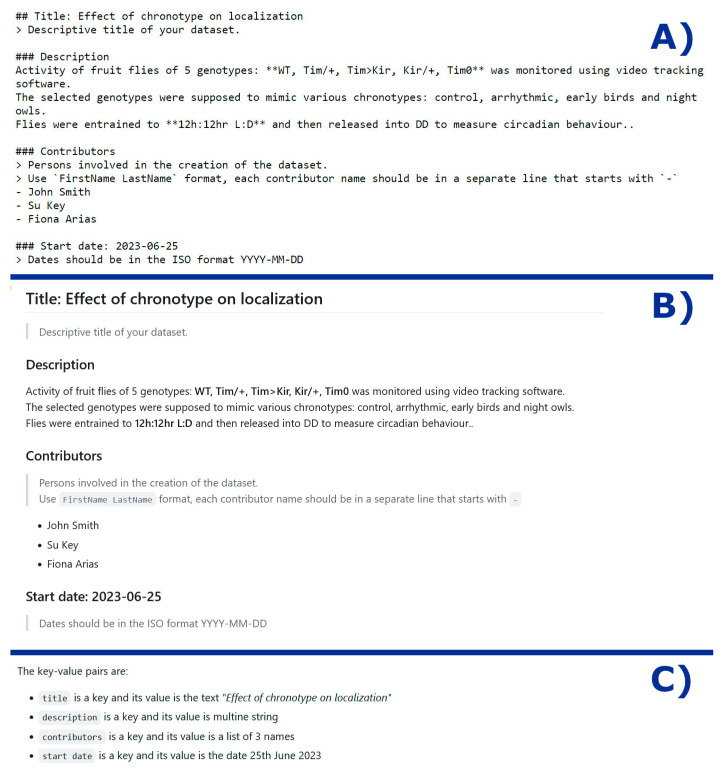
Proposed Markdown-compatible README template format. (**A**) Example of the README description seen as a simple text. (**B**) The same description file but rendered using the Markdown formatting. (**C**) The key-value pairs included in the description.

## Data Availability

The developed templates, guidelines, and discussions around them are openly available in a GitHub repository: https://github.com/circadianmentalhealth/circadian-data-standards (accessed on 25 August 2023).
